# The Role of Clock Genes in Fibrinolysis Regulation: Circadian Disturbance and Its Effect on Fibrinolytic Activity

**DOI:** 10.3389/fphys.2020.00129

**Published:** 2020-03-13

**Authors:** Pamela Carmona, Natalia Mendez, Carmen G. Ili, Priscilla Brebi

**Affiliations:** ^1^Instituto de Fisiología, Facultad de Medicina, Universidad Austral de Chile, Valdivia, Chile; ^2^Programa de Doctorado en Ciencias Médicas, Universidad de La Frontera, Temuco, Chile; ^3^Laboratory of Integrative Biology, Center for Excellence in Translational Medicine, Scientific and Technological Bioresource Nucleus (BIOREN), Universidad de La Frontera, Temuco, Chile; ^4^Laboratorio de Cronobiología del Desarrollo, Instituto de Anatomía, Histología y Patología, Facultad de Medicina, Universidad Austral de Chile, Valdivia, Chile

**Keywords:** non-communicable diseases, fibrinolytic system, PAI-1, clock genes, chronodisruption, prothrombotic phenotype

## Abstract

The fibrinolytic system is critical during the onset of fibrinolysis, a fundamental mechanism for fibrin degradation. Both tissue plasminogen activator (tPA) and urokinase plasminogen activator (uPA) trigger fibrinolysis, leading to proteolytic activation of plasminogen to plasmin and subsequently fibrin proteolysis. This system is regulated by several inhibitors; plasminogen activator inhibitor-1 (PAI-1), the most studied, binds to and inactivates both tPA and uPA. Through the action of plasmin, this system regulates several physiological processes: embryogenesis, activation of inflammatory cells, cell proliferation and death, synaptic plasticity, wound healing, and others. The deregulated intervention of fibrinolysis in the pathophysiology of various diseases has been widely studied; findings of altered functioning have been reported in different chronic non-communicable diseases (NCD), reinforcing its pleiotropic character and the importance of its physiology and regulation. The evidence indicates that fundamental elements of the fibrinolytic system, such as tPA and PAI-1, show a circadian rhythm in their plasmatic levels and their gene expression are regulated by circadian system elements, known as clock genes – *Bmal, Clock*, *Cry*-, and accessory clock genes such as *Rev-Erb* and *Ror*. The disturbance in the molecular machinery of the clock by exposure to light during the night alters the natural light/dark cycle and causes disruption of the circadian rhythm. Such exposure affects the synchronization and functioning of peripheral clocks responsible for the expression of the components of the fibrinolytic system. So, this circadian disturbance could be critical in the pathophysiology of chronic diseases where this system has been found to be deregulated.

## Circadian Rhythms, Clock Genes, and Their Close Relationship With Hemostasis and Fibrinolysis

The temporal organization of a living being is influenced by environmental stimuli and by internal biological clocks that are endogenously regulated in all living things. From an evolutionary point of view, events such as day and night, represented by light/dark cycles, were incorporated as a relevant time mark to predict environmental changes and as an anticipatory mechanism to perform and optimize activity/rest cycles (Mohawk et al., 2013). Circadian rhythms are intrinsic biological oscillations with a period close to 24 h; in mammals, they are driven by the circadian synchronization system (Albrecht, 2012). This system has a hierarchical organization; it is composed of the pacemaker or central clock, located in the suprachiasmatic nucleus, which is synchronized through the environmental light signals that proceed from the retina and are transported by the retinohypothalamic tract (Golombek and Rosenstein, 2010). The central clock, in turn, synchronizes peripheral clocks located in virtually all tissues and organs through nerve and/or endocrine signals (Schibler et al., 2015). Circadian rhythms are generated at cell level through transcriptional/translational loops (central and accessories loop). Interconnected and self-regulated by positive and negative feedback, these loops are composed for transcription factors collectively referred to as clock genes (Mohawk et al., 2013). The central loop is composed of the CLOCK:BMAL1 heterodimer, which promotes the rhythmic expression of the repressor proteins PER1-3 and CRY1-2 through E-box sites. Subsequently, PER and CRY translocate to the nucleus once they have been modified post-translationally, feeding back and limiting their own expression by displacing the CLOCK:BMAL1 from their promoter site. A cycle of this negative and positive feedback lasts approximately 24 h, thus generating circadian rhythmicity (Curtis et al., 2014; Takahashi, 2016).

The CLOCK:BMAL1 also promotes rhythmic transcription of the accessory loop components, such as the REV-ERBα/β nuclear receptor, which represses the transcription of the *Bmal1* gene by binding to the retinoid-related orphan receptor response element (RORE) in the promoter site of *Bmal1* (Partch et al., 2014; Honma, 2018). Other genes that structure the accessory loop are retinoid-related orphan receptors (RORs); *Ror*α/β/γ have been shown to activate the transcription of *Bmal1* through the binding of their respective proteins to RORE sites (Guillaumond et al., 2005). In addition, RORs modulate the expression of important components of the central loop such as CLOCK and CRY (Ueda et al., 2005; Takeda et al., 2012), which indicates that they are strongly involved in the regulation of the expression of clock genes and therefore of molecular machinery functions (Mazzoccoli et al., 2012a). The clock genes of the central and accessory loop regulate the rhythmic expression of other target genes called clock-controlled genes (CCGs), which in turn are related to multiple physiological functions such as behavior, metabolism, hemostasis, and immunity (Liu et al., 2008; Jetten, 2009; Shimba et al., 2011; Mavroudis et al., 2018). The central loop is also regulated by another accessory pathway, which includes the D-box albumin transcriptional activator binding protein (DBP), transcriptionally regulated through an E-box site, and the binding protein NFIL3, transcriptionally regulated through a RORE site. The DBP and NFIL3 proteins regulate positively and negatively, respectively the expression of genes that have D-box sites at their promoter site, such as *Per*, *Cry*, or *Rev-Erb* and other CCGs (Ueda et al., 2005; Curtis et al., 2014; Man et al., 2016; Mavroudis et al., 2018). Other data indicate that the mechanisms by which CLOCK:BMAL1 regulates the transcription of core clock genes do not apply to CCGs and suggest that the primary function of CLOCK:BMAL1 is to regulate the chromatin landscape at its enhancers to facilitate the binding of other transcription factors. This implies that CCG expression would be indirect, based on the interaction between the circadian clock and other signaling pathways (Trott and Menet, 2018; Beytebiere et al., 2019).

The circadian clock literally affects all physiological functions and behaviors, contributing significantly to the production and maintenance of endocrine rhythms modulating the levels of endocrine factors as well as the ability of the tissues to respond to these stimuli throughout the day (Richards and Gumz, 2013; Gamble et al., 2014; Challet, 2015). The evidence suggests that specific clock genes regulate different functions of the physiology of innate and adaptive immune cells (Silver et al., 2012; Casanova-Acebes et al., 2013; Pritchett and Reddy, 2015; Scheiermann et al., 2018), indicating that the regulation of immune response is under circadian control. Furthermore, the overall evidence shows that there is a mutual relationship: The clock controls some crucial metabolic pathways, and the metabolism feeds back to the clock machinery, synchronizing functions such as the production and expenditure of energy with the circadian patterns of the expression of metabolic genes in synchrony with the light/dark cycles, replenishing the proteins and enzymes during the resting phase that are needed to perform physiological functions in optimal conditions during the activity phase (Bellet and Sassone-Corsi, 2010; Mazzoccoli et al., 2012b; Masri et al., 2014). Moreover, circadian rhythms are important regulators of cardiovascular physiology; peripheral clocks are present in each of the types of cardiovascular cells, regulating various physiological functions such as endothelial function, blood pressure, and heart rate (Crnko et al., 2019). In relation to hemostasis, robust circadian oscillations in the number of circulating platelets and in the markers of platelet-endothelial aggregation and adhesion have been demonstrated (Scheer et al., 2011). A clear circadian expression of prothrombotic factors such as von Willebrand factor has also been shown, displaying maximum expression during the activity phase in humans and rodents, while on the other hand demonstrating a clear regulation of fibrinogen expression through clock genes (Somanath et al., 2011). Also, parameters of the coagulation system, such as prothrombin time (PT) and activated partial thromboplastin time (APTT), displayed a circadian rhythm, with the shortest PT being recorded late at night and early in the morning (Budkowska et al., 2019). All the expression profiles of circadian hemostasis previously described favor a prothrombotic phenotype when the circadian function of the molecular clock is deregulated.

Practically all tissues and organs have peripheral clocks, synchronized by powerful environmental signals such as light/dark cycles. Nowadays, modern society is further exposed to interruption of the synchrony of circadian rhythms through activities such as shift work, work at night, or chronic jet-lag, promoting a chronodisruption that has proven consequences for human health (Erren and Reiter, 2009, 2013). Therefore, it is not surprising to see that alterations in the circadian rhythm are involved in an increasing number of various chronic diseases, such as diabetes, obesity, chronic respiratory diseases, cardiovascular diseases (CVDs), and cancer (Haus and Smolensky, 2006; Scheer et al., 2009; Pietroiusti et al., 2010; Stevens et al., 2011; Plano et al., 2017). Interestingly, in several of these chronic diseases, the deregulation of the fibrinolytic system is also demonstrated in some of its components (Medcalf, 2007; Oishi, 2009; Godier and Hunt, 2013; Draxler and Medcalf, 2015; Svenningsen et al., 2017). In particular, an association between CVDs and an alteration in the levels of tissue plasminogen activator (tPA) expression and mainly plasminogen activator inhibitor-1 (PAI-1) has been demonstrated, the latter being studied as a possible marker of cardiovascular risk (Declerck and Gils, 2013; Tofler et al., 2016; Jung et al., 2018) and senescence (Eren et al., 2014; Yamamoto et al., 2014; Vaughan et al., 2017).

## Fibrinolytic Activity and Its Regulation Through Clock Genes

**FIGURE 1:**
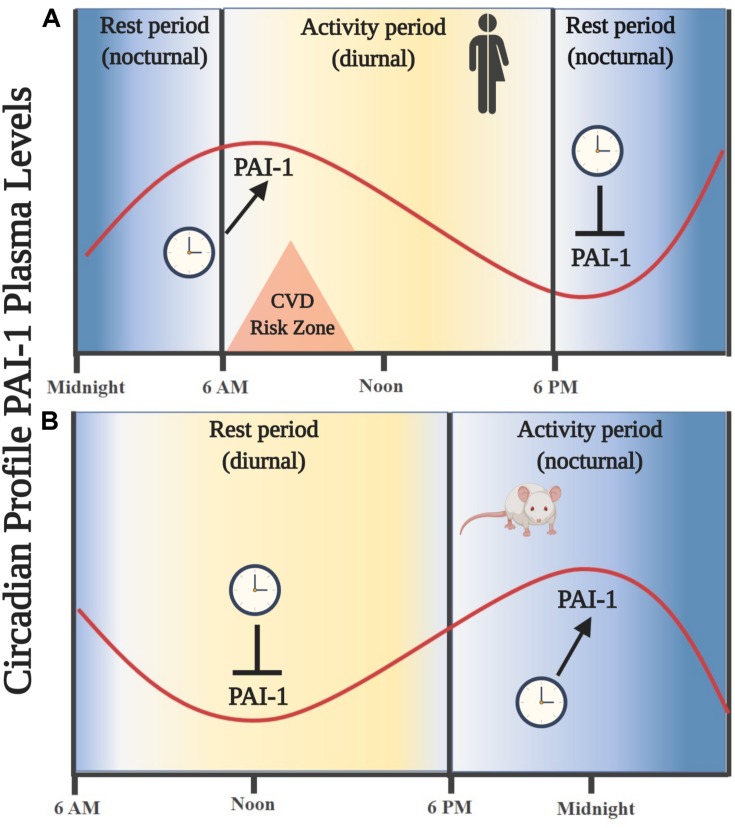
Oscillations in the circulating levels of PAI-1 are directed by an endogenous circadian synchronization system. **(A)** Circadian rhythm of PAI-1 in humans. An increase in circulating levels is observed leaving the resting phase (nocturnal), showing a maximum amplitude entering the activity phase (diurnal) during the first hours of the morning. **(B)** Circadian rhythm of PAI-1 in rodents. An increase in circulating levels is observed leaving the resting phase (diurnal), showing a maximum amplitude in the activity phase (nocturnal). Similarity is noted in the oscillation profile of circulating PAI-1 in the phase of activity/rest of both species; thus, a rodent model is useful for the study of key components of the fibrinolytic system.

**FIGURE 2:**
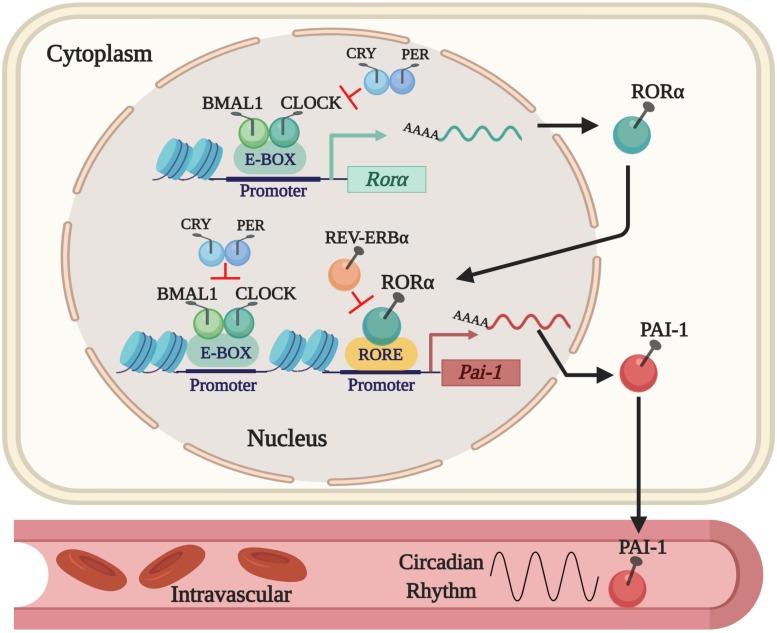
The current regulatory mechanism proposed for the expression of PAI-1 as a CCG. The expression of the *Pai-1* gene is positively regulated by the CLOCK/BMAL1 dimer that binds to E-box elements and by RORα that binds to RORE sites in its promoter site. Expression is repressed by displacing the CLOCK/BMAL1 dimer by PER/CRY and by REV-ERBα, which competes with RORα for the RORE sites in the PAI-1 promoter.

Thus far, we know that tPA and PAI-1 plasma levels oscillate robustly in circadian form in humans and rodents, decreasing and increasing, respectively, during the activity phase in both species, contributing to a state of hypofibrinolysis during this period (Angleton et al., 1989; Andreotti and Kluft, 1991; Ohkura et al., 2007; Scheer and Shea, 2014; Budkowska et al., 2019). This feature allows rodent models to be used for investigating the mechanisms that regulate fibrinolysis and its possible damage (see Figure 1). The rise in plasma PAI-1 levels during the beginning of the activity phase could explain the higher incidence of myocardial infarctions and strokes in humans in the morning (Pavlov and Ćelap, 2019). Experimental animal and cell models have shown that the expression of the *Pai-1* gene is directly regulated by clock proteins, which act as transcription factors either by stimulating or repressing its expression. In cell models, it has been shown that both heterodimers – CLOCK/BMAL1 and CLOCK/BMAL2 – promote the expression of the *Pai-1* gene through two E-box elements, located at its promoter site (Maemura et al., 2000; Schoenhard et al., 2003; Oishi et al., 2007). Moreover, CLOCK seems to be a positive regulator for the expression of the *Pai-1* mRNA because it has been found to decrease its expression levels and have no circadian rhythm when the *Clock* gene has been silenced by a small interfering RNA (siRNA) in endothelial cell culture and in mice (Cheng et al., 2011). The same occurs in a mutant mouse model for CLOCK (Ohkura et al., 2006) or when PER2 is overexpressed (Oishi et al., 2009). Regulation through other clock genes such as CRY1/2 has shown that they act as negative regulators in the expression of the *Pai-1* mRNA and also determine the characteristic plasma oscillatory pattern of PAI-1, because an increased and arrhythmic plasmatic expression have been observed in CRY1/CRY2^–/–^ mice (N. Ohkura et al., 2006). In addition, the *Pai-1* transcript is promoted by RORα and repressed by REV-ERBα by binding to response elements related to the orphan receptor (RORE sites) at its promoter site (Wang et al., 2006). These clock genes (through their proteins RORα and REV-ERBα) regulate the expression of CLOCK:BMAL1 and other CCGs (Mohawk et al., 2013; Curtis et al., 2014; Takahashi, 2016). In fact, the evidence described above demonstrates that *Pai-1* is a CCG (see Figure 2). There are other important transcriptional regulations, such as the sirtuins (SIRTs), which modulate the expression of various clock genes in a circadian manner, repressing transcription through their histone deacetylase (HDAC) activity and counteracting the CLOCK histone acetylase (HAT) protein activity, which in turn promotes the expression of clock genes and CCGs (Bellet and Sassone-Corsi, 2010), thus balancing the transcriptional activity of the circadian system. There is also evidence to suggest that *Pai-1* may be epigenetically modified through a mechanism that involves SIRT1, a class III chromatin histone deacetylase (SIRTUIN1), promoting heterochromatin formation and *Pai-1* gene silencing, specifically by direct acetylation of histone 4 lysine 16 (H4K16) at its promoter site (Lopez-Legarrea et al., 2013; Wan et al., 2014).

## Discussion

### Disturbance in the Molecular Machinery of the Clock: Its Effect on Thrombogenesis and Fibrinolysis Through PAI-1

As described above, the fibrinolytic system is an important endogenous defense system against intravascular thrombosis. The evidence indicates that its main modulator is the PAI-1 inhibitor, which is currently classified as an independent risk factor for CVD (Tofler et al., 2016; Jung et al., 2018). On the other hand, the circadian expression of PAI-1 is regulated by the molecular machinery of peripheral clocks; various clock genes determine both their level of expression and circadian rhythmicity. This suggests that an alteration in the expression of the clock genes, by means of genetic ablations DNA mutations or by circadian disruption by alterations of the dark/light cycles, could promote a decrease in fibrinolytic activity or hypofibrinolysis, thereby increasing the predisposition to the development of CVD.

In recent years, it has been reported that disruption models of the *Bmal1* clock gene in mice develop various characteristics that combined describe a prothrombotic phenotype. One study that used mice deficient in *Bmal1* (*Bmal1*^–/–^) showed significant differences from the control group: shorter times of cessation of tail bleeding, significantly shorter arterial occlusion times after an injury, increased plasma fibrinogen levels and a significant increase in plasma levels, and an absence of a circadian pattern for PAI-1 (Somanath et al., 2011). Other models have confirmed the progression of a prothrombotic state in knockout mice (KO) for *Bmal1* during the development of aging: The results showed shorter prothrombin times, increased platelet count, decreased endothelial production of nitric oxide and thrombomodulin expression (Hemmeryckx et al., 2011), confirming a close functional relationship between the central loop of the clock and the regulation of the hemostasis. Later studies also showed that *Bmal1* deficiencies (*Bmal1*^–/–^ mice) clearly disrupt the daily rhythm in the expression of relevant coagulation and fibrinolysis factors. Specifically in the liver, an increase in the gene expression of *fibrinogen*, *tissue factor*, *protein C*, and *Pai-1* have been observed while *tPA* is decreased; however, plasma levels of PAI-1 are reduced, which disagrees with reports by Somanath et al., where they were found to be increased. This discrepancy may be related to differences in the light/dark protocols in which the mice were kept (Somanath et al., 2011; Hemmeryckx et al., 2019).

The effects found in the liver are interesting because this organ is the most important in the synthesis of coagulation and fibrinolysis factors (Dimova and Kietzmann, 2008; Leebeek and Rijken, 2015). Furthermore, it has often been described as an important peripheral circadian clock (Reinke and Asher, 2016; Tahara and Shibata, 2016; Zwighaft et al., 2016); therefore, any desynchronization or disruption of its circadian rhythm could have side effects on the physiological functions it performs.

On the other hand, CLOCK is also important for maintaining the diurnal variation of thrombogenesis. The mutation of CLOCK in mice (CLOCK^mut^) alters the fibrinolytic system; total and active plasma levels of PAI-1 are elevated and tPA is reduced. In addition, these effects would be related to the small but significant increase in vascular occlusion time observed in this experimental model (Westgate et al., 2008). Interestingly, in patients with acute coronary syndrome, CLOCK and PAI-1 were overexpressed in peripheral blood macrophage cells, suggesting that CLOCK might play an important role in the progression of atherosclerotic plaques (Jiang et al., 2018). Taken together, these results show that clock genes control the expression of key components in hemostatic function and the fibrinolysis system, which leads to an increased risk of developing a prothrombotic phenotype and thus an increased risk of harmful cardiovascular events.

### Update on Unusual Exposure to Artificial Light and Its Impact on Fibrinolytic Activity

A reduced fibrinolytic activity due to an increase in the expression of PAI-1 is a characteristic risk factor for CVD due to its role in vascular homeostasis (Mavri et al., 2004; Oishi, 2009). In addition, as described above, its plasma levels have a typical circadian rhythm and its gene expression is regulated by clock genes; therefore, PAI-1 is a component capable of being disturbed through a circadian disruption. It is known that the light is a dominant stimulus for training circadian rhythms in mammals, and exposure to light at inappropriate times such as during the resting stage could alter the physiology of tissues, organs and systems (Roenneberg et al., 2013; Plano et al., 2017), including the fibrinolytic system. To date, the studies that are known have examined the experimental effect of a chronic alteration of the photoperiod on the expression of PAI-1. For this, mouse models have been used (rodents and humans have the same circadian profile for PAI-1 and tPA in the rest/activity cycle), exposed to a temporary desynchronization of the endogenous circadian clock, imitating the working schemes of rotating shifts. The results show that the hepatic expression of *Pai-1* and its plasma levels increase significantly while the *tPA* expression decreases. Additionally, increased levels of plasma corticosterone were found, suggesting a relationship between the hypothalamic–pituitary–adrenal axis and the expression of *Pai-1* (Oishi and Ohkura, 2013). In another study, alterations in the hepatic expression of clock genes such as *Clock*, *Bmal1*, and *Per1* were found in mice exposed to artificial light pulses during the night phase, and in the same way, an increase in both transcriptional hepatic expression and plasma protein of PAI-1 was observed (Aoshima et al., 2014). In humans, it seems that the indispensable requirement for an increase in PAI-1 levels is a chronic circadian desynchronization because short-term circadian misalignment lowers the 24-h PAI-1 levels (Morris et al., 2016).

## Conclusion

The evidence suggests that a disturbance of the endogenous circadian rhythm mediated by the photoperiod alteration induces a state of hypofibrinolysis, mainly by promoting an alteration in the expression of PAI-1 and tPA, due to its direct regulation by the circadian synchronization system. Additional studies on animal models and humans are needed to determine the association of these findings on the origin and/or development of CVD and other chronic diseases in which PAI-1 and tPA are involved in their pathophysiology, in order to prevent the appearance of chronic diseases in adulthood due to exposure to chronodisruption.

## Author Contributions

PC wrote the first draft and developed the final version of the manuscript; NM, CI, and PB conducted a critical review for important intellectual content. All authors contributed to the manuscript revision, read, and approved the submitted version.

## Conflict of Interest

The authors declare that the research was conducted in the absence of any commercial or financial relationships that could be construed as a potential conflict of interest.

## Abbreviations

CCG: clock-controlled gene
CVD: cardiovascular disease
NCD: non-communicable disease
PAI-1: plasminogen activator inhibitor-1
ROR: retinoid-related orphan receptor
RORE: ROR-response elements
tPA: tissue plasminogen activator.
